# Malignant Perivascular Epithelioid Cell Tumor of the Uterus

**DOI:** 10.7759/cureus.41685

**Published:** 2023-07-11

**Authors:** Gurneel K Dhanesar, Harish Rengarajan, Baidarbhi Chakraborty

**Affiliations:** 1 Internal Medicine, New York Medical College, Denville, USA; 2 Internal Medicine, New York Medical College, Saint Mary's General Hospital, Passaic, USA; 3 Pathology, Saint Clare Hospital, Denville, USA

**Keywords:** nab-sirolimus, metastatic disease, tsc1, mtor inhibitors, small bowel pecoma, pecoma, gynecology oncology, oncology

## Abstract

Perivascular epithelioid cell tumors (PEComa) are soft tissue tumors. They belong to the family of mesenchymal tumors and include angiomyolipomas, clear cell sugar tumors of the lung, and PEComas not otherwise specified (NOS). Tuberous sclerosis complex 1 (TSC1) and tuberous sclerosis complex 2 (TSC2) gene mutation is associated with PEComa, which causes hyperactivation of the mammalian target of rapamycin (mTOR) signaling pathway. In some cases, transcription factor E3 (TFE3) gene fusion is also observed. They are usually found in middle-aged women with clinical symptoms of abnormal uterine bleeding and pelvic pain. Radical surgical resection with clear margins is the mainstay of the treatment. We encountered a 54-year-old woman who had postmenopausal abnormal uterine bleeding. A hysterectomy was planned, but pelvic adhesions were discovered during the procedure. As a result, she underwent an exploratory laparotomy with hysterectomy, appendectomy, and total omentectomy. The biopsy of the uterus, left ovary, and a small bowel nodule revealed diffuse growth of epithelioid cells with eosinophilic granular cytoplasm with HMB45 staining, which indicated PEComa. A treatment plan with an mTOR inhibitor nab-sirolimus was proposed for the patient. Early detection, a multidisciplinary approach, and timely treatment are crucial for better disease prognosis.

## Introduction

Perivascular epithelioid cells are mesenchymal tumors composed of distinctive perivascular epithelioid cells with immunoreactivity to melanocytic and smooth muscle markers. Immunohistochemistry is reported to have positive (HMB-45 and Melan-A) and smooth muscle (actin and desmin) markers. Perivascular epithelioid cell tumors (PEComa) are a family of mesenchymal neoplasms that include angiomyolipoma, clear cell "sugar" tumors of the lung, and lymphangiomyomatosis [1]. PEComa family tumor incidence is high in patients with tuberous sclerosis complex (TSC), a genetic disorder that is autosomal dominant with incomplete penetrance [2]. The diagnosis is mainly based on histopathological and immunohistochemical analysis. Radical surgical resection is associated with a better prognosis. We present a case of a 54-year-old woman with abnormal uterine bleeding and no medical history of tuberous sclerosis diagnosed with uterine PEComa with small bowel involvement.

## Case presentation

A 54-year-old woman visited the outpatient clinic complaining of postmenopausal uterine bleeding, abdominal pain, and sore back. She has a history of iron deficiency anemia but no history of tuberous sclerosis. A first-degree relative had melanoma. She has no history of smoking or alcohol consumption.

On the physical examination, she had normal vital signs, a soft abdomen that was non-tender and non-distended, and no palpable lymph nodes or masses were appreciated. Her laboratory findings were significant for hemoglobin of 9.7g/dL (reference range: 13.5-17.5 g/dL), mean corpuscular volume 74.7 fL and total leucocyte count of 23.5 x10*3/uL (reference range: 3.5-10.5 × 10*3 µL), platelet count 395 x 10*3/uL (reference range: 150-450 x 10*3/uL), human chorionic gonadotropin (hCG) 7 mIU/ml (reference range: 0-5 mIU/ml), alanine aminotransferase 17 U/L (reference range: 9-46 U/L), aspartate aminotransferase 46 U/L (reference range: 10-36 U/L) and a total bilirubin of 0.3 mg/dL (reference range: 0.2-1.2 mg/dL), albumin 2.1 g/dl (reference range 3.6-5.2 g/dl). On the ultrasound of the abdomen and pelvis examination, it was discovered that the patient's uterus was enlarged. However, an endometrial biopsy did not reveal any abnormalities. 

**Figure 1:**
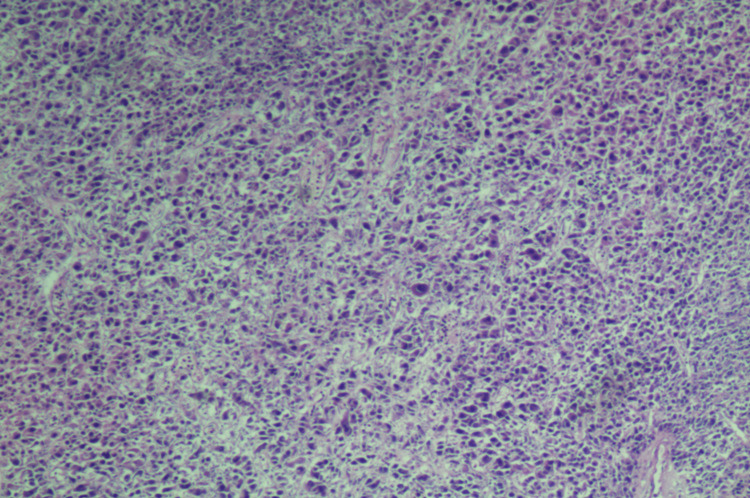
Diffuse growth of epithelial cells with eosinophilic granular cytoplasm; low magnification 40x

**Figure 2:**
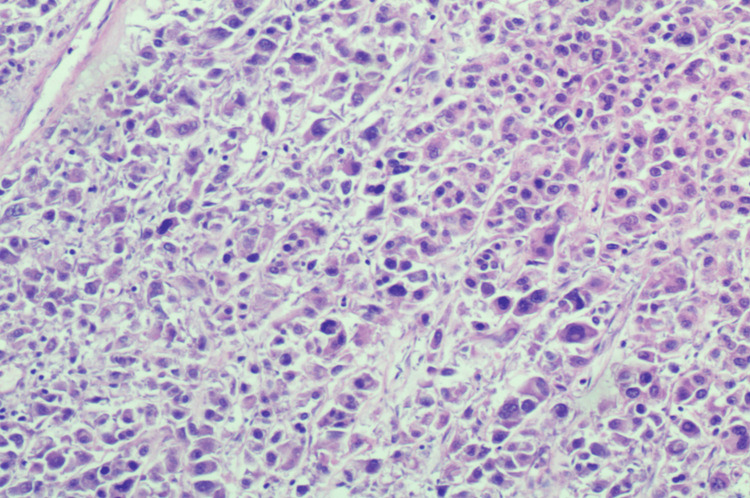
Intermediate magnification 100x

**Figure 3:**
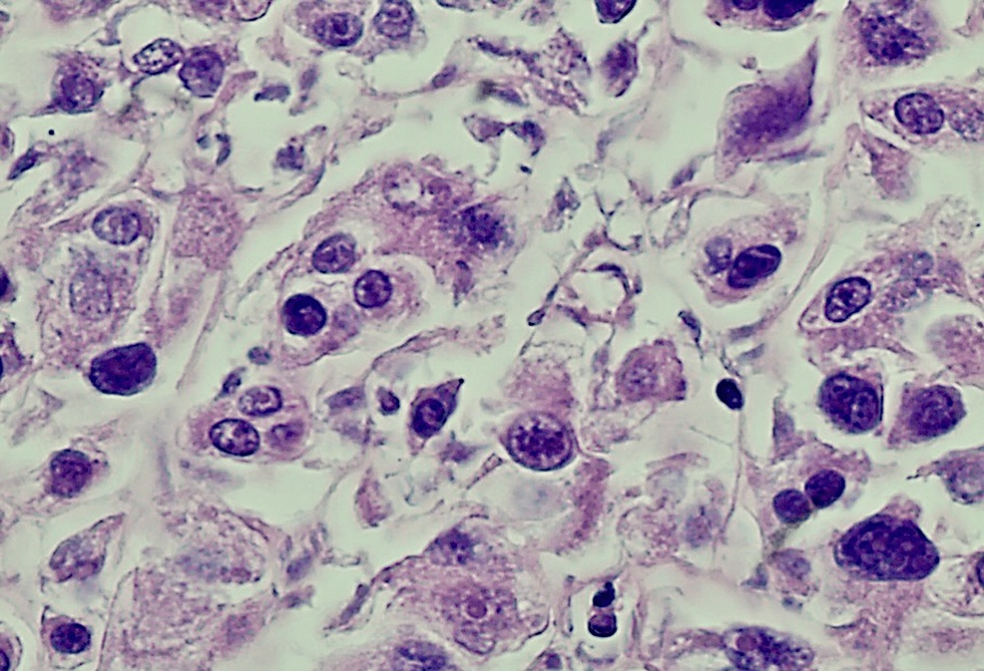
High magnification 400x

**Figure 4:**
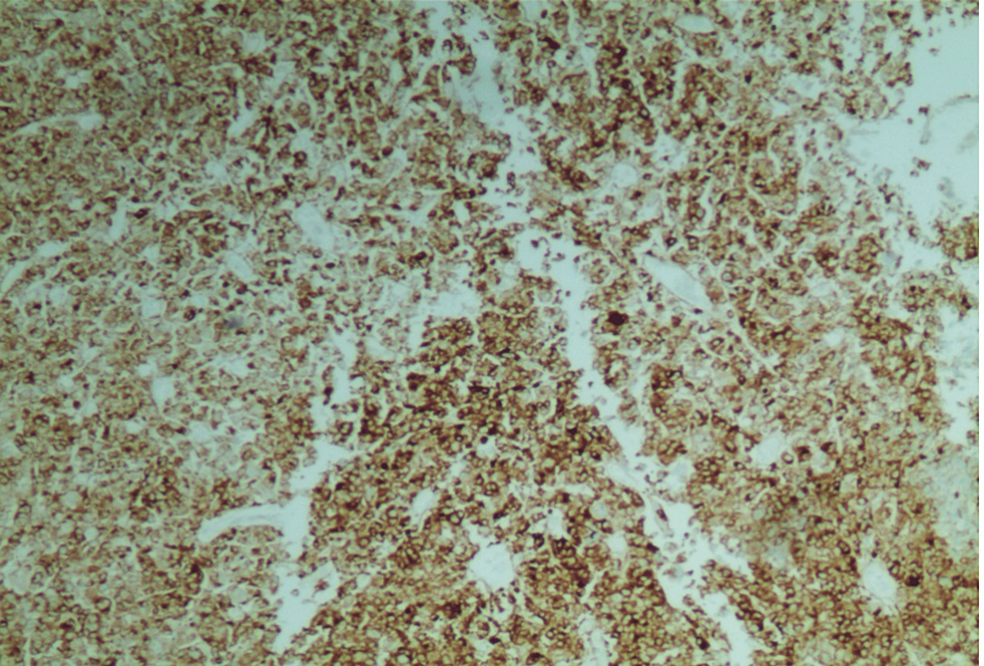
HMB45 immunostain; strong and diffuse staining

The patient was scheduled to undergo a hysterectomy and bilateral salpingo-oophorectomy, but the procedure was complicated due to extensive pelvic adhesions. The uterus, small bowel nodule (the antimesenteric border of mid-small bowel), ovary, and peritoneum were then sent for pathological analysis. Upon examination, the uterine serosa showed gray-tan soft hemorrhagic tissue with multiple adherent red-brown blood clots, the left ovary had friable gray-tan hemorrhagic necrotic appearing tissue, and the small bowel nodule measured 0.4x 0.3x 0.3 cm irregular fragment of rubbery gray-tan tissue. The histology of the uterine serosa, small bowel nodule, and left ovary revealed diffuse growth of epithelioid cells with eosinophilic granular cytoplasm (Figures 1-3) with a mitotic index of 4/10 HPF and lymph-vascular invasion. However, the peritoneum showed no epithelioid cells with eosinophilic granular cytoplasm. The results of the immunohistochemistry test showed that the tumor was positive for desmin, HMB45 (Figure 4), MutL protein homolog 1 (MLH1), MutS homolog 2 (MSH2), MutS homolog 6 (MSH6), PMS2, and phosphatase and tensin homolog (PTEN), while negative for S-100, SMA, caldesmon, SOX10, melanocyte-inducing transcription factor 1 (MITF-1), and equivocal for transcription factor E3 (TFE3). The molecular genetic testing revealed tuberous sclerosis 1 (TSC1) pathogenic variant in exon 17. The diagnosis of PEComa in stage T3aN0M0 was established. The treatment with mTOR inhibitors was proposed because of the TSC1 gene mutation and equivocal TFE3 equivocal staining. She has already completed two cycles of nab-sirolimus, and further debulking treatment will be based on her response to the current therapy.

## Discussion

Perivascular epithelioid cell tumors are rare mesenchymal neoplasms with histological findings of spindle and epithelioid cells with eosinophilic to clear cytoplasm. The family of these tumors includes various tumors such as renal and extrarenal angiomyolipomas, a clear-cell sugar tumor of the lung, primary extrapulmonary sugar tumor, clear-cell myomelanocytic tumor of the falciform ligament/ligamentum teres, a primary cutaneous PEComa, and a PEComa not otherwise specified (NOS) are also part of this family [1].

Most cases are reported in the fourth decade of life, predominately in the female sex. The organs involved are the uterus, falciparum ligament, and retroperitoneum, lung, breast, cardiac septum, kidney, and gastrointestinal involvement is reported in the liver, rectum, small and large bowel [2,3].

Patients typically exhibit clinical symptoms of unusual uterine bleeding and pelvic pain. While imaging studies can detect the presence of masses, they are not very effective in distinguishing PEComa from other types of tumors. Therefore, extensive histopathological and immunohistochemical analyses are required to establish a diagnosis. Histologically, they are characterized by epithelioid and spindle cells. The architecture ranges from sheets, nests, cords, and trabeculae, with nuclei ranging from transparent to eosinophilic [4]. In immunohistochemistry and molecular analysis of 32 patients with uterine PEComas at Massachusetts General Hospital, the incidence of positive HMB-45, cathepsin K, Melan-A, and MiTF was expressed in 83%, 93%, 77%, and 79%, respectively. One muscle marker was noted in every case, including smooth muscle actin, desmin, and h-caldesmon. Only 20% of PEComas are reported to be positive for TFE3 nuclear staining [5]. 

The tumor grade is evaluated based on size, mitotic rate, cellularity, necrosis, and vascular invasion. Aggressive tumors are characterized by a size above 5 cm, a high mitotic rate of >1/50 HPF, increased cellularity, coagulative necrosis, and vascular invasion [6].

Radical surgical resection is considered the mainstay of treatment. The other proposed treatment is with mTOR inhibitors due to mutations in tuberous sclerosis complex (TSC) genes TSC1 and TSC2. The TSC gene products negatively regulate mTOR, a protein complex associated with cellular growth and synthesis [7]. TFE3 gene testing should be considered in patients with malignant uterine PEComas as therapy with vascular endothelial growth factor, or phosphatidylinositol-3-kinase, is more effective in these patients. In our patient, treatment was also proposed with mTOR inhibitors due to TSC1 gene mutation and equivocal TFE3 equivocal staining.

## Conclusions

This case highlights the importance of a comprehensive and multidisciplinary approach for diagnosing PEComa, i.e., testing for genetic and immunochemistry markers. Genetically targeted therapy is more effective, and thus, overall survival can be improved, and better prognosis in patients with malignant perivascular epithelioid tumors can be achieved. 
